# Fast Small‐Angle X‐Ray Scattering Tensor Tomography: An Outlook into Future Applications in Life Sciences

**DOI:** 10.1002/smtd.202500162

**Published:** 2025-07-16

**Authors:** Christian Appel, Margaux Schmeltz, Irene Rodriguez‐Fernandez, Lukas Anschuetz, Leonard C. Nielsen, Ezequiel Panepucci, Tomislav Marijolovic, Klaus Wakonig, Aleksandra Ivanovic, Anne Bonnin, Filip Leonarski, Justyna Wojdyla, Takashi Tomizaki, Manuel Guizar‐Sicairos, Kate Smith, John H. Beale, Wayne Glettig, Katherine E. McAuley, Oliver Bunk, Meitian Wang, Marianne Liebi

**Affiliations:** ^1^ Center for Photon Science Paul Scherrer Institute Villigen 5232 Switzerland; ^2^ Department of Physics Chalmers University of Technology Gothenburg SE‐41296 Sweden; ^3^ ARTORG Center for Biomedical Engineering Research Universität Bern Bern 3008 Switzerland; ^4^ Department of Otorhinolaryngology Head and Neck Surgery Inselspital University Hospital and University of Bern Bern 3010 Switzerland; ^5^ Institute of Materials École Polytechnique Fédérale de Lausanne (EPFL) Lausanne 1015 Switzerland; ^6^ Institute of Physics École Polytechnique Fédérale de Lausanne (EPFL) Lausanne 1015 Switzerland; ^7^ Department of Otorhinolaryngology Head and Neck Surgery Lausanne University Hospital (CHUV) and University of Lausanne (UNIL) Lausanne 1011 Switzerland; ^8^ Institute for Biomedical Engineering ETH Zürich Zürich 8092 Switzerland; ^9^ Center for Scientific Computing, Theory and Data Paul Scherrer Institute Villigen 5232 Switzerland; ^10^ The Sense Innovation and Research Center Lausanne and Sion Lausanne 1007 Switzerland

**Keywords:** hierarchically structured materials, incus, nanostrucutral orientation, ossiculoplasty, saxs, tensor tomography

## Abstract

Small Angle‐X‐ray Scattering Tensor Tomography (SAS‐TT) is a relatively new but powerful technique for studying the multiscale architecture of hierarchical structures particularly relevant to life science applications. Currently, the technique is very demanding on synchrotron beamtime, which limits its applications, especially for cases requiring a statistically relevant number of samples. This study reports the first SAS‐TT measurement at a macromolecular X‐ray crystallography beamline, PX‐I at the Swiss Light Source (SLS), with an improvement in acquisition time from 96 h/Mvoxel in the pilot experiments to 6 h/Mvoxel with comparable sampling, defining a new standard for fast SAS‐TT with a micrometer beam size and allowing to record a full tomogram in 1.2 h. Measurements are performed on the long and lenticular process of the incus bone, one of the three human auditory ossicles. The main orientation and degree of alignment of the mineralised collagen fibrils are characterised, as well as the size and shape of the mineral particles which show relevant variations in different tissue locations. The study reveals three distinct regions of high fibril alignment, most likely important pathways of sound throughout the ossicular chain, and highlights the technique's potential to aid in future developments in middle ear reconstructive surgery.

## Graphical abstract

This study reveals the future potential of SAXS tensor tomography at macromolecular X‐ray crystallography beamlines and life sciences applications. A new standard in fast data acquisition is set, allowing for up‐to 10x higher throughput. The measurements show mineralised collagen fibrils within the incus bone of the human auditory ossicle.

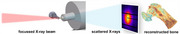

## 1 Introduction

The development of X‐ray diffraction, scattering, and imaging techniques at third‐generation synchrotron radiation facilities allowed researchers to visualise materials across multiple length scales. Macromolecular crystallography^[^ ^1^ ^]^ (MX) has revolutionised our understanding of life at the molecular level by providing 3D structures of biomolecules at atomic resolution <1 Å and is today widely used by both academia and the pharmaceutical industry. In comparison, Small‐Angle X‐ray Scattering (SAXS) and Wide‐Angle X‐ray Scattering (WAXS, also called X‐ray diffraction XRD) probe nanoscale features across multiple length scales ranging from less than 1 Å to hundreds of nm. This includes samples from hard and soft matter in aqueous solutions or solid forms.^[^ ^2^, ^3^, ^4^, ^5^, ^6^ ^]^ Scanning the sample with a tightly focused X‐ray beam and analysing its diffraction patterns, a technique called scanning SAXS/WAXS or µSAXS/WAXS, allows visualising localised structural heterogeneity across macro‐structures in life and materials science applications.^[^ ^7^ ^]^ Scanning SAXS/WAXS can bridge length scales across macroscopic samples and offers statistical information on nanoscale features volume averaged over the X‐ray beam and step size. In life sciences, structures with hierarchical order and structures on well‐defined length‐scales are well suited to be studied with scanning SAXS. Examples include collagen, abundant in the human body, e.g. as part of the extracellular matrix of tissues, myelin sheets wrapping around neurons, or myofilament as a constituent of muscle tissue.^[^ ^8^, ^9^, ^10^, ^11^, ^12^, ^13^, ^14^ ^]^ Scanning SAXS/WAXS can be combined with computed tomography and extended to tensor tomography to include the orientation information about nanostructure^[^ ^13^, ^15^, ^16^, ^17^, ^18^ ^]^ or crystalline texture.^[^ ^19^, ^20^, ^21^, ^22^ ^]^ A recent review summarises their application to the structure of hierarchical biological materials.^[^ ^23^ ^]^

SAS‐TT was first developed in 2014/2015 at the cSAXS beamline of theSLS at the Paul‐Scherrer‐Institute (PSI).^[^ ^24^, ^25^ ^]^ Computer tomography concepts are combined with scanning SAXS to retrieve the full 3D reciprocal space map, from which one can retrieve information about the average nanostructure within each voxel including its orientation. For biomedical research, this 3D nanoscale information can be directly linked to structural properties and provide important structural insights on hierarchically structured materials, for example, the structure of hard bone tissue based on abundance and orientation of calcified collagen or to reveal myelination levels, integrity, and axon orientations in a mouse brain.^[^ ^26^, ^27^, ^28^ ^]^ As for other synchrotron methods, SAS‐TT is limited to biopsy or post‐mortem samples. While the technique is still under development, multiple synchrotrons (ESRF, MAX IV, PETRA III, DIAMOND, NSLS‐II, and SSRL4) are commissioning or have already commissioned its use at some of their beamlines. A limiting factor for the technique's application and accessibility to a broader user community is related to time‐consuming measurements, which can take more than 24 h for a single sample. Such long measurement times are directly related to the technique's capabilities of probing the 3D reciprocal space map of a macroscopic sample, typically millimeters in size. For this purpose, a few hundred 2D projections are taken with a few thousand diffraction patterns each. To make SAS‐TT relevant to a wider user base, particularly for life‐science applications in which experiments are required to be performed on a statistically relevant number of samples, the acquisition speed of a full data set needs to be improved significantly.

The required experimental setup comprises two translational and two mutually perpendicular rotational motions, as well as a 2D X‐ray detector. A typical SAS‐TT measurement of a 1.5 × 2.5 mm^2^ sample at 25 µm spatial resolution requires ≈300 2D projections with 6000 detector images for each 2D projection. For this case, a fully sampled SAS‐TT tomogram amounts to 1.8 M detector images, each having typically 2 to 16 M pixels of ≈16–32 bits, in total ≈8–20 TB disk usage per dataset which must be recorded in a reasonable time. A comparative quantity for measurements at different resolutions and sample sizes is the time needed to measure 1 M voxels, this comparison is valid only if the sampling, i.e., number of projections compared to resolution and sample dimensions are similar and was calculated with the tomogram size spanning the full field of view. The first SAS‐TT experiment in 2014 at cSAXS took 35.5 h, corresponding to 96 h/Mvoxel at a voxel size of 25µm^3^. Since then, multiple beamlines have started to implement the technique, and there is a growing demand for considerably reducing acquisition time to boost the applicability and impact of this novel method. For sub‐micron beam size (500 nm), Frewein et al.^[^ ^22^ ^]^ recently demonstrated a significant improvement in measurement efficiency using the fast detector readout capabilities of an Eiger X 4 M at 500 Hz.

The essential limiting factor for the measurement time per sample is the scanning speed, determined by X‐ray beam flux, scanning stages, the frame rate and read‐out of the X‐ray detector, and their synchronization. The overall increase in flux density at 4^th^ generation synchrotrons will allow faster and finer scanning of samples which in return means however that the deposition rate of the X‐ray dose on the sample increases, leading to potential temperature increase and/or resulting in damage of the sample during the measurement. X‐ray‐induced heating can be mitigated by carrying out measurements under cryogenic conditions, which also significantly reduces X‐ray‐induced radiation damage, spreading from the atomic to the nanoscale, thus allowing investigations of radiation‐sensitive soft materials. The desired instrumentation improvements are met at the macromolecular crystallography beamline PX‐I at SLS, which has been optimised for high‐throughput cryogenic crystallography including features such as a micro focused X‐ray beam with high flux, a multi‐axis goniometer – SmarGon for multi‐orientation data collection,^[^ ^29^, ^30^ ^]^ a large area and fast readout X‐ray detector – EIGER 16 M for low‐noise data acquisition,^[^ ^31^ ^]^ the implementation of fast 2D continuous scans for serial synchrotron crystallography,^[^ ^32^, ^33^ ^]^ automation from cryogenic sample exchange, software assisted sample alignment with possibility to easily adjust the rotation centre combined with an on‐axis optical microscope and suitable online computing resources for fast feedback on the measurements. A helium filled flight‐tube and a semi‐transparent beamstop were added to reduce air scattering and record beam transmission for transmission corrections.

In this work, we demonstrate the capabilities and future perspective of SAS‐TT at a state‐of‐the‐art macromolecular crystallography (MX) beamline. The measurements reveal the potential for high‐throughput SAS‐TT experiments, demonstrate for the first time a SAS‐TT measurement under cryogenic conditions, fast software‐alignment allowing for automation, and report a record in data acquisition for a full tomogram measured in 1.2 h, respectively at 6 h/Mvoxel at a voxel size of 25µm^3^, 15 times faster than the first measurements in 2014.

## 2 Results

**Figure 1:**
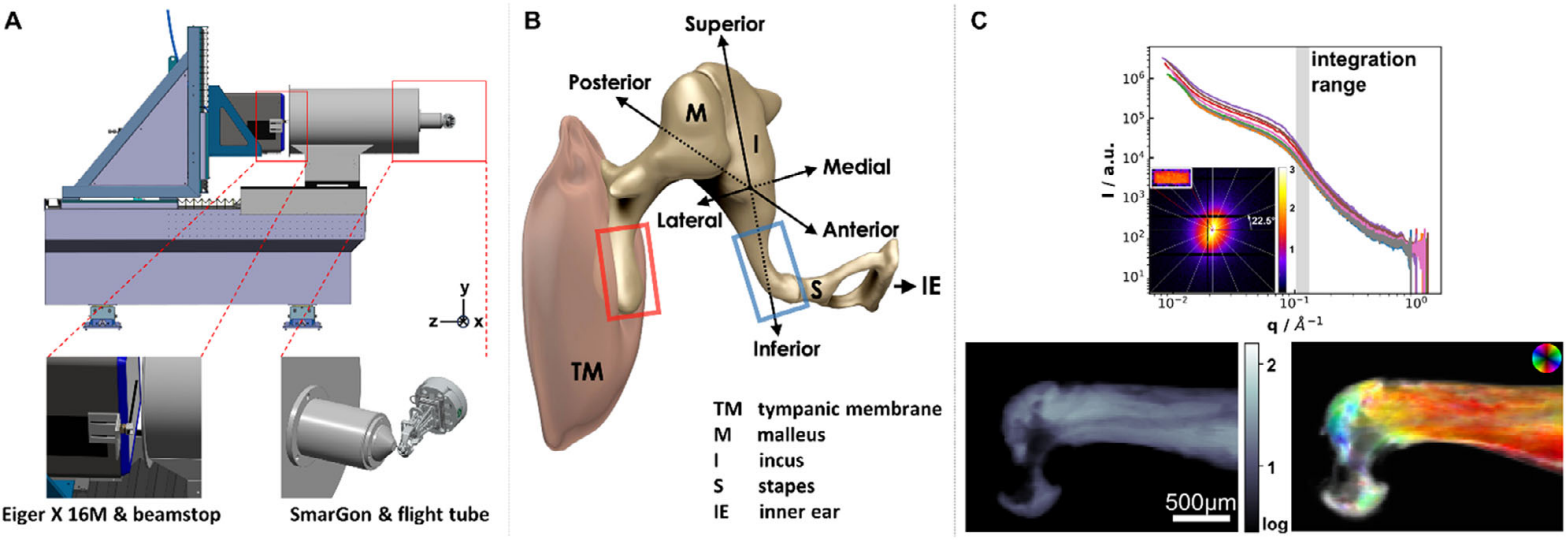
A) Drawing of the experimental setup with custom‐built flight tube, SmarGon, and Eiger X 16M detector together with semi‐transparent beamstop. B) 3D view of the middle ear (adapted from "Radiopaedia‐Drawing Middle ear ossicles and tympanic membrane‐no labels" at AnatomyTOOL.org by Frank Gaillard, license: CC BY‐NC‐ND) to show the tympanic membrane and ossicular chain (malleus, incus, and stapes). Within the small b/w sketch shown in the inset, the blue and red boxes highlight the regions that were scanned for the incus and malleus respectively. C) Segment‐wise integrated 1D scattering curves of the incus with an inset of the corresponding 2D scattering pattern, together with 2D absorption and the orientation and abundance plot below. For the orientation and abundance plot, the intensity is integrated within the *q* range indicated with a grey box in the 1D intensity plot and analysed as described in the main text. Here, hue shows the direction of anisotropy, the value shows the symmetric intensity, and the saturation the degree of orientation.

The content of this work is structured in two parts, for which two samples from the human auditory ossicle, the malleus and incus bone, as shown in **Figure** **1**, are used. In the first part, we will focus more on technical aspects related to fast SAS‐TT data acquisition and instrumentation aspects. We included a brief study on photon statistics on the malleus to explore how much further fast acquisition schemes can be pushed in the future. In the second part (3.Application Example), we will present a pilot study on the long and lenticular process of the incus to reveal the potential of SAS‐TT as a tool to investigate samples for the life science community.

### 2.1 Instrumentation and Control System

The required instrumentation for a SAS‐TT setup comprises stages for fast raster scanning, two rotational degrees of freedom, a low‐noise large area detector with fast readout capabilities, and a control system with optimised motor motion. The state‐of‐the‐art setup at PX‐I is very well suited to perform SAS‐TT measurements. It is an undulator beamline equipped with a Si (111) double crystal monochromator capable of operating at energies between 6 and 20 keV. A two‐stage focusing with a dynamically bendable mirror and a Kirkpatrick‐Baez mirror pair allows for variable beam size from 5 × 5 µm^2^ to 80 × 80 µm^2^ at the sample position 28 m from the source. These focusing capabilities determine the spatial resolution of the SAS‐TT measurements for which the volume averaged information of the nanoscale can be recorded. Furthermore, PX‐I offers some application features designed for macromolecular crystallography under cryogenic conditions which include an automated sample exchange, cryogenic storage of samples, and a so‐called cryojet for sample cooling during measurement. These features offer opportunities to extend studies on perishable, frozen, and radiation sensitive samples as well as fully automated data acquisition schemes for high‐throughput studies.

The SmarGon (SmarAct GmbH)^[^ ^30^ ^]^ allows XYZ positioning and three angles of rotation around an arbitrary point in space. Its positioning resolution is <5 nm. The SmarGon is combined with Aerotech stages with motion and control overheads as little as ≈0.4 s in between lines for continuous scanning in *snake motion* (alternatingly forth and back scanning of lines). Therefore, all conditions for optimised acquisition schemes are well met with the given setup. The available range for scanning is 5 × 5 mm^2^, 0…360° and 0…45° for the rotational degrees of freedom (φ and χ). The technical rendering in Figure 1A shows the setup that facilitates the fast scanning at various orientations of the sample as required for proper sampling of the 3D reciprocal space as needed for the SAS‐TT reconstructions. As an example of its overall acquisition performance, a sample with dimensions of 2.8 × 1.6 mm^2^ measured at 20 µm resolution and a frame rate of 80 Hz took 16 h at PX‐I with 13 h being the total exposure time. This illustrates the highly efficient use of synchrotron light at this beamline. The measurement was performed at 12.4 keV, resulting in a dose of 8.22 MGy on the sample. Details about acquisition parameters and dose estimations can be found in Section S1 (Supporting Information).

The beamline's large area detector, the DECTRIS EIGER X 16 M, allows acquisition rates up to ≈133 Hz with 16 M active pixels, and a region of interest (ROI) mode with 4 M active pixels up to 750 Hz. In contrast to regular MX experiments, the detector was placed at 1.2 m distance from the sample to resolve the smaller scattering angles with a custom‐built flight tube filled with helium placed in between the sample and detector to reduce air scattering and absorption. The sample‐detector distance was calibrated using silver behenate (AgBH) powder as a reference.^[^ ^34^ ^]^ A 3 × 3.5 mm^2^ (diameter, thickness) silicon single crystal was installed as a semi‐transparent beamstop on a motorised stage in front of the EIGER reducing the intensity of the direct beam to a level that is not saturating the detector, allowing for a simultaneous transmission measurement. More details about the transmission measurement can be found in Section S2 (Supporting Information).

Another factor contributing to the highly efficient use of the synchrotron‐radiation beamtime is the on‐axis microscope in combination with the graphical user interface, developed for fast and precise alignment of crystals and the setting up of grid‐scans for raster scanning across samples.^[^ ^32^, ^33^ ^]^ While this is in place at most state‐of‐the‐art MX beamlines, scanning SAXS and SAS‐TT beamlines are typically designed for versatility rather than automation. Therefore, software and hardware support for sample alignment is typically less optimized and takes correspondingly much longer – on the order of hours rather than minutes at PX‐I.

Fast data pipelines with analysis and live feedback capabilities are crucial for an efficient and well‐controlled measurement. We deployed a pipeline adapted from the cSAXS MATLAB package^[^ ^35^ ^]^ to provide online feedback on data acquisition, and the computing resources at PX‐I were capable to keep up with the measurements in real time for all acquisition rates. We computed 16 segments from the raw detector image and reduced them to 8 assuming inversion symmetry of the scattering signal. In Figure 1C,D SAXS profiles are shown for the 8 segments. The scattering signal in each segment can be associated with information about the nanostructure with a specific orientation within the sample. In the case of bone, we use the range of *q* = 0.5–0.6 nm^−1^ to quantify the content and orientation of the bio mineralised crystals within collagen fibrils of the bone following the procedure described by Bunk et al.^[^ ^36^ ^]^ The main direction of orientation for the nanoscale features is extracted from the phase of the cosine θ and the degree of orientation is calculated by dividing the asymmetric intensity a_1_ (amplitude of the cosine) by the symmetric intensity a_0_ (average intensity). The orientation and scattering of mineralised collagen can be mapped based on transmission normalised scattering features in each individual pattern and visualised as a pixelated image with help of the color‐coding according to the color wheel. Here, the hue shows the direction of anisotropy (main orientation), the value shows the symmetric intensity, and additionally, the saturation shows the degree of orientation. Highly oriented material appears with bright colors whereas low‐oriented material appears in a tone from black to grey, depending on its abundance.

### 2.2 Perspective for Data Acquisition at 4^th^ Generation Synchrotrons

In recent years, several synchrotrons have been constructed and upgraded to 4^th^ generation such as MAX IV in Sweden, Sirius in Brazil, and the ESRF in France, getting much closer to a diffraction‐limited storage ring. The SLS 2.0 upgrade^[^ ^37^, ^38^ ^]^ started in October 2023, which includes upgrades of the insertion device and X‐ray optics for the PX‐I beamline. The beamline is expected to obtain an increase in brilliance by up to two orders of magnitude and potentially 3 orders in terms of overall flux density with a wide bandwidth monochromator. It is unquestionable that a flux‐hungry technique such as SAS‐TT strongly benefits from this upgrade. However, the increase in flux must be matched with appropriate instrumentation and software for fast data acquisition. We benchmarked the experiment control and instrumentation setup of PX‐I with the available flux of 2.56 10^12^ photons/s (calibration performed with glassy carbon standard, GCL14^[^ ^39^ ^]^).

**Figure 2:**
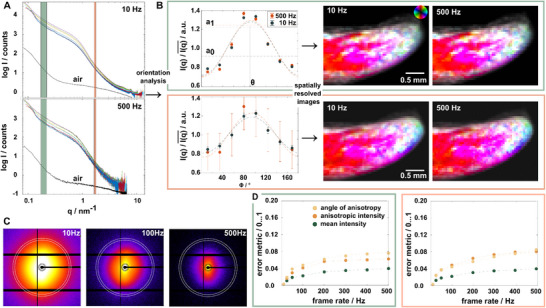
This figure illustrates the analysis flow for a scanning SAXS experiment, i.e. a single projection of a SAXS tensor tomography dataset. A,C) present the raw data, once in the form of 2D detector data for 10, 100, and 500 Hz, and the 1D scattering curves for 8 integrated segments of the diffraction pattern after averaging regularly spaced opposing segments. The analysis is performed on 2 selected *q* ranges (high and low q) which are highlighted by the green and orange boxes in the 1D scattering curves (A), and the white dashed rings in the raw detector frames (C). The intensity within this region is integrated and an orientation analysis is performed as a function of the azimuthal angle. An example plot for such an analysis is shown on the left in B). A sinus function is fitted to the data for each point of the 2D raster scan and used to compute a colorful image as described in the main text. Four images from two measurements (10, 500 Hz) of the bone are shown on the right with nanostructural features color‐coded according to the circular legend. These measurements were done at 8 frame rates ranging from 10…500 Hz, which are now used to perform a similarity analysis for both *q* ranges using all points of the grid scan (error metric described in main text). Results are shown in D) with dashed lines acting as guides for the eye.

The increase in photons and higher dose can also lead to radiation‐induced damage of the sample, which means that acquisition schemes must be adjusted accordingly. To explore the effect of photon statistics on a relevant sample, we varied the dose on the sample by measuring 2D projections of the malleus from the human ossicular chain with a range of different acquisition rates between 10 and 500 Hz covering a 50‐fold increase in surface dose on the sample, 3.58 10^4^ Gy for 500 Hz to 1.79 10^6^ Gy for 10 Hz. Measurements were performed starting with 10 Hz and their results are shown in **Figure** **2**. With increasing frame rate, photon counts on the detector reduce visibly in the raw data, see diffraction patterns in Figure 2C, as well as in the radially integrated 1D SAXS curves, see Figure 2A. Measurements above 133 Hz were performed in the ROI mode with only 4 M active pixels in the centre and therefore have a reduced *q*‐range. Scattering maps of the orientation of nanostructures from the bone are calculated from the azimuthal plots for two different *q*‐ranges, *q* = 0.17…0.22nm^−1^ and *q* = 1.65…1.77 nm^−1^, to explore low and high photon count statistics from the sample.

We choose the integration width in *q* for both ranges small to be representative for nanostructural features with a narrow width, i.e., diffraction‐like peaks, and to remain comparative to bin sizes used for the *q*‐resolved SAS‐TT reconstructions. For 500 Hz, the larger error bars for larger *q* are related to reduced counting statistics. We can extract the mean intensity a_0_, anisotropic amplitude a_1_, and the direction of the anisotropic scattering θ to present them in the color‐coded images as shown in Figure 2B. To compare measurements at different frame rates in a quantitative manner, we use the normalised root‐mean‐square error E as a measure for the similarity of the mean intensity and the anisotropic amplitude of the images *I(x_i_,y_j_)* with the 10 Hz measurement as the reference *R(x_i_,y_j_)*:^[^ ^40^, ^41^ ^]^

(1) $$E=\sqrt{\frac{\sum_{x,y}{\left|I\left(x,y\right)-R\left(x,y\right)\right|}^{2}}{\sum_{x,y}{\left|R\left(x,y\right)\right|}^{2}}}$$

For the angle of the anisotropic intensity as the main orientation of the nanostructure, we use the square‐root of 1 minus the dot product of the image and reference. Results of our error metric are shown in Figure 2D, with 0 meaning that two images are identical whereas 1 means that there is no similarity between the images. For all three quantities, we observe a slow increase of the error. This trend is within our expectations as the number of photons decreases with increasing frame rate. Overall, the error remains below 8% even at 500 Hz acquisition. We see the same trend and similar quantitative values for both *q* ranges. This means that the orientation analysis is robust enough against the noise variation in the data.

With the upcoming upgrade to SLS 2.0 and the advent of new detector technology such as the Jungfrau^[^ ^42^ ^]^ or Matterhorn^[^ ^43^ ^]^ detector, measurements in the 1–10 kHz regime are within reach. Already now, we were able to record a full SAS‐TT dataset of the malleus bone at 500 Hz with 153 projections, a field of view of 1.2 × 2 mm^2^ at 25 µm resolution and under cryogenic conditions. For more details about acquisition parameters and dose calculation, please check Sections S1 and S3 (Supporting Information) with results of the reconstruction shown in Figure S1 (Supporting Information). The full dataset was recorded in 1.2 h including all overheads, corresponding to 6 h/Mvoxel. Fast acquisition texture tomography experiments using a sub‐micron focused beam of 0.5 µm and an Eiger X 4 M detector has recently been demonstrated by Frewein et al.^[^ ^22^ ^]^ with 500 Hz and 6 h measurement time, calculating with the full field of view this accounts for 1.7 h/MVoxel, which would need to be corrected by a different sampling due to a larger amount of air included due to the sub‐micro‐beam and goniometer constraint on alignment. In direct comparison, the larger sample extension and 50 times larger voxel‐size reported here impose different hardware requirements such as the need for faster scanning stages, while the larger voxel size allow for an accurate pre‐alignment which minimizes the field of view around the sample.

In addition to fast acquisition of projections, the two‐axis SmarGon goniometer combined with the on‐axis optical microscope and implemented software‐assisted alignment allows for efficient sample changes, reducing the time between mounting and starting a tomogram to ≈10 min. Alignment procedure can be automized similar than done for protein crystals, and the available sample changing robot can be used. For a detailed overview of the most impactful changes leading to improved acquisition speed, we direct interested readers to Section S4 (Supporting Information).

## 3 Application Example: Nanoscale Survey of a Human Auditory Ossicle

The application of SAS‐TT to hierarchical structures offers unique insights across multiple length scales, as illustrated in the following pilot study on a human incus, one of the three auditory ossicles in the middle ear. The ossicular chain, see Figure 1B, is composed of the malleus, incus, and stapes and is responsible for the sound transmission between the external environment and the inner ear, where the sound is transformed to an electrical signal. The impedance difference between the air and the fluid‐filled cochlea is matched by the ossicles. Interestingly, studies suggested that the auditory ossicles are already fully developed at birth, with only little bone remodelling happening in a lifespan, in order to preserve the ossicular architecture and ensure a stable sound transmission throughout life.^[^ ^44^ ^]^ Other studies correlated bone mineral density with sound transmission, showing that a remodelling process lowering the mineral density could happen in regions experiencing more motion and higher forces.^[^ ^45^ ^]^ It has also been shown that the degree of mineralization and apatite orientation was higher in the ossicles than in long bones.^[^ ^46^ ^]^ One recent study using Synchrotron‐based X‐ray Phase Contrast Imaging (SR X‐PCI) identified a vast vascular network inside the ossicles with associated perivascular zones of less bone density.^[^ ^47^ ^]^

Pathologies of the middle ear such as chronic otitis media, otosclerosis, or cholesteatoma usually affect ossicle integrity, resulting in hearing loss. These conditions often require reconstructive surgery of the ossicular chain. Therefore, gaining insights on the multi‐scale architecture of the ossicles, the orientations of the collagen/mineral components, and the location of potential bone‐remodelling regions is crucial to improve surgery planning, as well as the design and placement of passive middle ear implants. While a part of the malleus was measured demonstrating fast SAS‐TT (Figure S1, Supporting Information) at 500 Hz with a surface dose of 3.58 10^4 ^Gy per projection (5.48 × 10^6^ Gy in total), the long and lenticular processes of the incus were measured at 83 Hz with a surface dose of 3.36 10^5 ^Gy (1.03 10^8 ^Gy), thus higher photon statistics and analyzed in detail as outlined below. With the higher brilliance of 4^th^ generation sources, photon statistics will be of sufficient quality for a full analysis in the fast mode.

### 3.1 Incus Measurement and Reconstructions

The 2.8 × 1.6 mm^2^ large sample was scanned with a 20 × 20 um^2^ micro focused X‐ray beam and pixel size and 83 Hz (0.012s) exposure time. In total, 306 projections were recorded for a total of 7 sub tomograms, respectively seven tilt angles (0…45°) to properly sample the 3D reciprocal space. Please check Section S1 (Supporting Information) for more details on acquisition parameters.

The fully integrated SAXS signal was used to align the projections using a Filtered Back Projection (FBP) algorithm from the cSAXS **MATLAB** package.^[^ ^35^ ^]^ Results of the alignment are shown in Figure S2 (Supporting Information). Reconstruction of the 3D reciprocal‐space map for specific q‐ranges is carried out using the software package MUMOTT^[^ ^48^ ^]^ (*version 0.2.1*) as described in Nielsen et al.^[^ ^49^ ^]^ The orientation of the main intensity for each voxel is determined from the eigenvector associated with the smallest eigenvalue of the rank‐2 tensor derived from the degree‐2 component of the spherical function's polynomial expansion. The degree of orientation is calculated as the ratio between the standard deviation (anisotropic component) and the mean (isotropic component). More details are available in Section S5 (Supporting Information).

### 3.2 Variation of Size and Shape of the Mineral Nanoparticles

The primary building blocks of bone are collagen fibrils reinforced with small mineral particles of hydroxy‐apatite. Amount, size, orientation, and distribution of these particles play a pivotal role in defining the mechanical properties of the bone‐collagen‐mineral composite.^[^ ^50^, ^51^ ^]^ Mineralization changes throughout the life cycle of bone^[^ ^14^ ^]^ as well as within regions where different forces or motions are applied.^[^ ^45^ ^]^ A low level of mineralization is found in newly formed bone and can be an indicator of sections where bone remodelling occurs within the ossicles. To extract information on the mineral particles, we use the T‐parameter model which is an indicator of the volume‐to‐surface ratio of the mineral particles without shape assumptions^[^ ^52^ ^]^ following the procedure described in Pabisch et al.^[^ ^51^ ^]^ We note that within this simplified model, a reliable readout of the T‐parameter is limited to the region for power‐law exponent ≤ 2. In bone, where the dimensions of the mineral and their spacing is in the same size scale, ambiguity between shape change or arrangement exist.^[^ ^53^ ^]^ However, changes in the slope of the scattering curve do indicate differences in the structure and/or structural arrangement of the mineral phase and have been reported to vary, for example, close to Ti‐implants^[^ ^54^ ^]^ or at the neurocentral growth plates.^[^ ^55^ ^]^ We included here a power‐law exponent ($I\;∼\;q^{-G}$) fit^[^ ^11^ ^]^ of the investigated particles in order to study the variation over the ossicle bone. The model is fitted to the *q* resolved reconstruction of the mean intensity to compute the invariant *q* from *q*_min_ = 0.025 to *q*_max_ = 0.28 nm^−1^, the Porod constant for 0.16 to 0.28 nm^−1^ and the power‐law exponent for 0.025…0.055 nm^−1^ from the background subtracted data. Radiation damage checks were performed after each sub‐tomogram, see Figure S3 (Supporting Information). These measurements indicate that the low *q* intensity is slightly increasing after the first sub‐tomogram. We focus our analysis on the local variations within the ossicle bone rather than interpreting quantitative values of the power‐law exponent.

**Figure 3:**
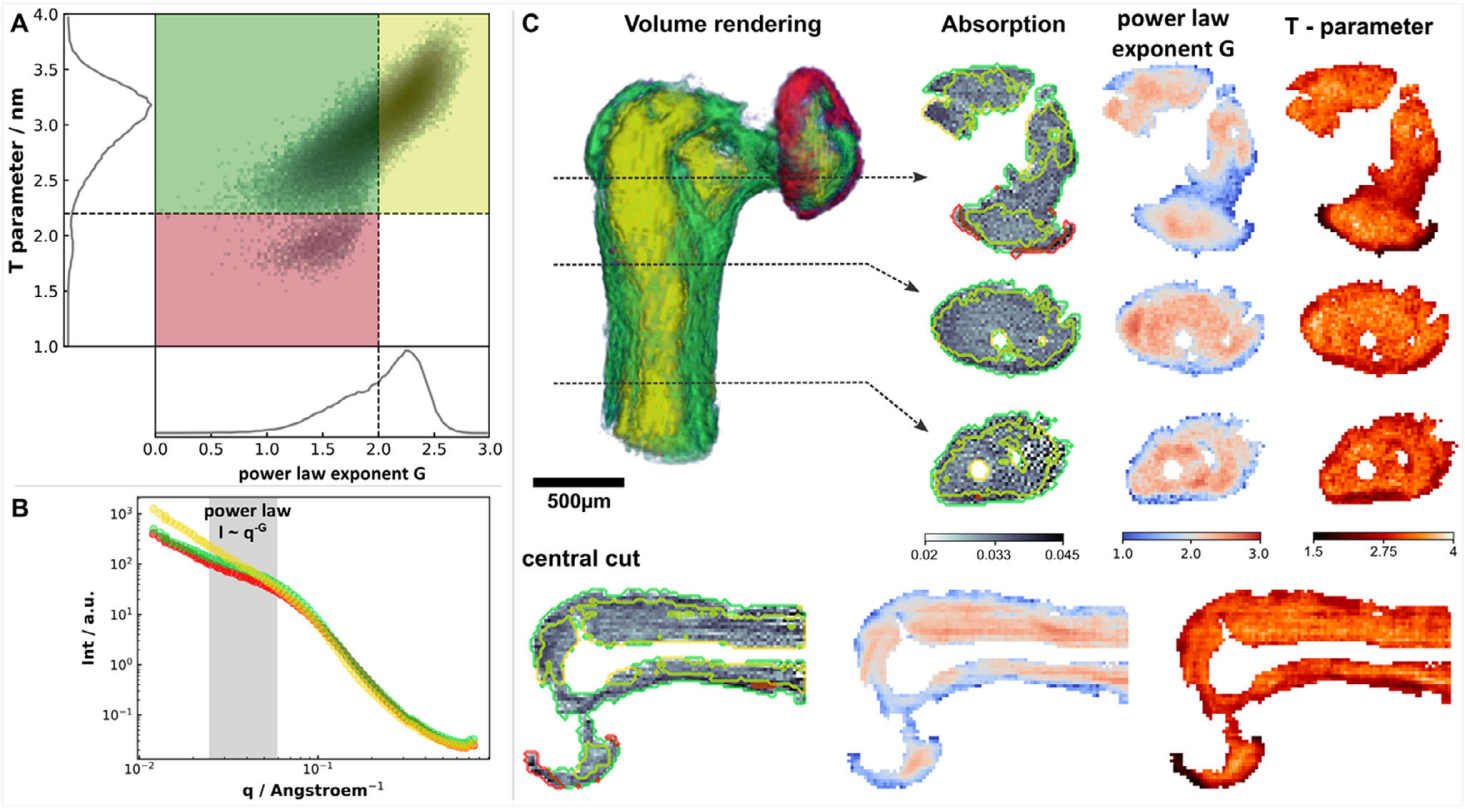
Q resolved the SAS‐TT of the incus. A) 2D histogram of T‐parameter and power‐law exponent. Data is segmented based on the histogram in 3 phases (red, green, and yellow), see details on the segmentation in the main text. Representative 1D scattering curves for each phase are shown in B), with the region used to calculate the power‐law exponent highlighted in grey. In C), a 3D volume rendering of the segmented long and lenticular processes of the incus is shown as well as slices from different cuts through the volume showing absorption, power‐law exponent, and T‐parameter. Contour lines color‐coded for the segmented phases are plotted on top of the slice for the absorption contrast.

The analysis of mineral particles is shown in **Figure** **3**. The power‐law exponent and mineral thickness (T‐parameter) is computed for all voxels of the 3D reconstructed volume masked with a thresholded absorption tomogram to avoid inclusion of volume outside of the main body. Figure 3A shows the distribution of the T‐parameter and the power‐law exponent values over all the voxels of the reconstructed volume, in the form of a 2D histogram. In the histogram, we clearly see a population of particles shifted to larger power‐law exponent values. This has been experimentally observed for immature bone, e.g. embryonic long bone mineralization in mice^[^ ^8^ ^]^ or healing bone close to the interface of biodegradable implants.^[^ ^11^ ^]^ From a scattering point of view this indicates a more compact shape of the mineral particles. Based on the distribution in the 2D histogram, which shows a small secondary side maximum around G≈1.7, and the theoretically highest valid G exponent of G = 2, we decided to classify our data into three phases based on the following two boundaries of G = 2 and T = 2.2 nm. For each segmented phase, a representative 1D scattering curve is shown in Figure 3B, while the spatial distribution of the phases is highlighted in the volume rendering in Figure 3C. The segmentation clearly separates the bone into the inner long process (yellow), an interfacial layer surrounding the long process and forming the bony pedicle (green), and an annular thin layer spreading around the lenticular process (red), highlighting the advantage of the multi‐contrast images obtained from SAS‐TT. Different slices through the 3D volume are shown for three parameters: absorption, power‐law exponent, and T‐parameter. The latter two are nanostructure features extracted from small‐angle scattering. The main vascular channel of a branched network known to exist inside the long process of the incus can be identified already in the absorption contrast.^[^ ^47^ ^]^ These channels are surrounded by the yellow phase with values of the power‐law exponent G >2. We also observe a correlation with an increased T‐parameter, but as mentioned, this calculation is no longer valid due to constraints of the model. The phase bordering the long process (green), is more pronounced in the inner lateral part rather than the anterior and posterior sides. The bony pedicle, which is bridging the long process to the lenticular process, consists almost exclusively of this green phase characterised by G <2 but larger T‐parameters. Focussing on the lenticular process, we can see a circular layer in red, which is characterised by significantly lower T‐parameters.

### 3.3 Orientation of Mineralised Collagen Fibrils

**Figure 4:**
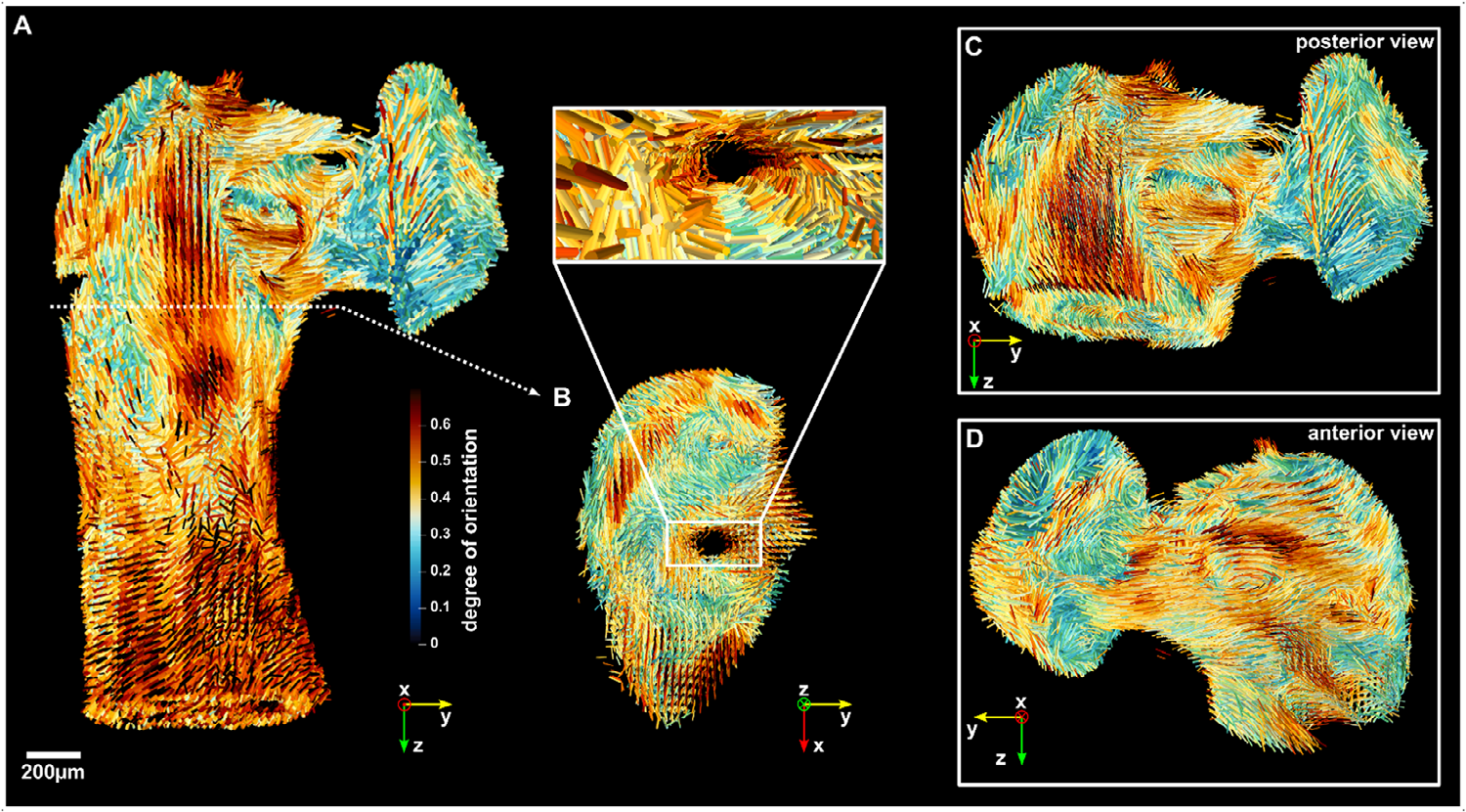
A) 3D glyph representation of the orientation of mineralised collagen fibrils within the long and lenticular process of the incus bone derived from SAS‐TT (q range 1.61…1.7 nm^−1^). The cylinder size is scaled with the mean intensity while the color represents the degree of orientation. C,D) highlight the bony pedicle with lenticular processes attached from the posterior and anterior view, while B) shows a cross‐section with a view through the long process. The expanded view along the large vascular channel within the long process illustrates the alignment of collagen fibrils close to the channel.

On the mesoscale, the SAXS diffraction pattern of collagen fibers are composed of two distinct signals: the meridional and equatorial reflections.^[^ ^56^ ^]^ Bundles of mineralized collagen fibrils form collagen fibers and thereby allow us to extract the main fibril orientation from the reconstructed data (more details in Section S7, Supporting Information). Fiber orientation was extracted from the scattering between 1.61…1.7 nm^−1^. **Figure** **4** illustrates the results in the form of a 3D glyph representation with cylinders pointing in the direction of the main structure's orientation contained in one voxel. The size of the cylinders is scaled with the mean intensity of the rank‐2 tensor, while the color represents the degree of orientation calculated from the standard deviation of the same tensor. Figure 4A–D shows different views of the lenticular and long process of the incus using the same 3D glyph representation (see coordinate systems).

The degree of orientation of the mineralised collagen fibrils seems to be higher in the long process compared to the lenticular process of the incus. The long process has a more regular and elongated shape, as well as an inner vascular network which could be the reason that the mineralised fibrils point into a dominant direction. The region of the bony pedicle, that makes the junction between the 2 processes, particularly visible in Figure 4C,D (posterior and anterior view), is clinically critical, as it is a non‐vascularised region. The bone is nourished by diffusion from the bony pedicle to the lenticular process, which is compromised in the case of acute or chronic inflammation. We notice bundles of fibrils highly oriented toward the lenticular process that build three bridges or pair of bridges between the long process and the bony pedicle connecting it to the lenticular process. Toward the lenticular process, the three bridges merge into a single path where fibrils appear to be more randomly oriented again as indicated by the brighter color.

**Figure 5:**
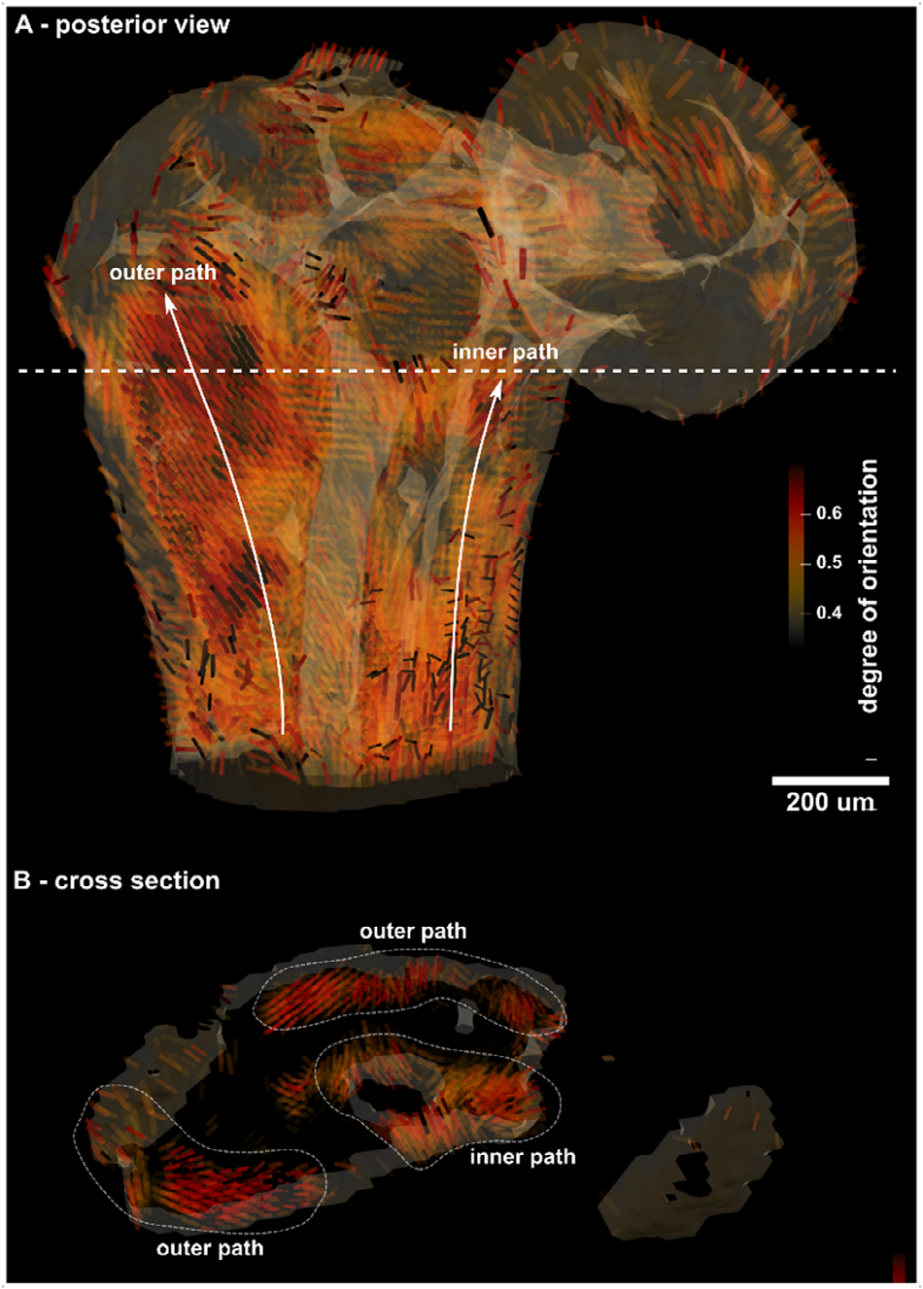
3D glyphs superimposed with an isosurface rendering of the absorption tomogram. Fibrils with a degree of orientation below 0.4 are fading out due to a strong transparency mask that is applied. A) posterior view of the long and lenticular process, the two white lines with an arrow highlight the outer path and inner path extending from the long process to the bony pedicle. B) shows a cross‐section extracted from the height highlighted by the dashed white line. The 3 regions with 2 outer and 1 inner path are additionally encircled with dashed lines and tagged.

Along the long process, we can observe the existence of 3 main paths showing a high degree of orientation of the mineralised fibrils. These paths are highlighted in the cross‐section shown in Figure 4B: two outer paths aligned along the long process, and one wrapping around the main vascular channel, also visible in Video S1 (Supporting Information). These three bundles are also illustrated in the posterior view in **Figure** **5** together with a cross section (5). Figure 5 is a combination of masking out fibrils of low anisotropy with a strong transparency mask for values <0.4, superimposed with an isosurface rendering of the absorption tomogram. Video S2 (Supporting Information) gives a better understanding of the 3D arrangement within this view, which supports the existence of these highly oriented paths that bridge the long process and the lenticular process via the bony pedicle. We note that the outer paths appear to be less anisotropic when they wrap around the top of the long process, however, this observation may be attributed to having multiple fibril directions superimposed within single voxels.

Highly anisotropic features within the ossicles might play a role in the mechanical properties and thereby affect efficient sound transmission. Morris et al.^[^ ^45^ ^]^ found lower bone mineral density in the joint connecting the lenticular process of the incus to the stapes. They believe this to be caused by biomechanical stress in response to sound conduction. With this in mind, we can trace back the 3 paths from the joint and thereby identify a potential route of sound propagation along the long process of the incus bone. Therefore, our findings could be highly interesting for future surgical planning, as they provide insights into collagen fibril orientation within bone, aiding in reconstructive strategies for the ossicular chain. One concrete example would be the optimization of the attachment points of a passive middle ear implant onto the long process of the incus. Additionally, our findings might also become relevant to modelling approaches for the human middle ear,^[^ ^57^, ^58^ ^]^ since these studies typically assume that it is not necessary to consider anisotropic mechanical properties for the ossicles.^[^ ^59^ ^]^

## 4 Conclusion

In this study, we show the potential of a state‐of‐the‐art macromolecular X‐ray crystallography beamline to perform fast SAS‐TT for life science applications. The beamline's setup is very well suited for quick alignment, fast data acquisition, and excellent data quality. In addition, the readily available cryogenic conditions may play a pivotal role for radiation sensitive samples. We investigated two samples from the human auditory ossicles, the incus and the malleus. Based on measurements of the malleus at different frame rates, we benchmarked the data quality in terms of different photon statistics and the future potential of SAS‐TT that is ready to leverage from the increase in photons at 4^th^ generation synchrotron sources. Already with the current photon flux of 2.56 10^12^ photons/s, we were able to acquire a full SAS‐TT dataset with 1.2 × 2.0 mm^2^ field of view, 25 µm resolution, and 153 projections at different rotational degrees of freedom in 1.2 h. This corresponds to 6 h/Mvoxel which is 15x faster compared to the first SAS‐TT measurements in 2015 with similar sampling conditions and the same voxel size. With the upcoming upgrade program SLS 2.0 and new detector technology, such as the Jungfrau or Matterhorn, data acquisition in the 1–10 kHz range will be in reach.

In the second part of the paper, a pilot study on the long and lenticular processes of the incus highlights the technique's potential impact for studies within the life science community. The analysis focuses on the distribution and content of mineral particles, the building unit of mineralised collagen fibrils, as well as their orientation. Collagen organisation is linked to the bone's mechanical properties and can therefore be used as an indicator for understanding the path of sound waves through the bulk of the auditory ossicle. Our analysis reveals that three distinct phases exist in the incus with different shapes and sizes of the mineralised nanoparticles. We are further able to extract the main orientation of the mineralised collagen fibrils and their anisotropy, which allows us to identify certain regions in the long process that exhibit a higher alignment. Here, we find two outer and one inner path along the largest vascular channel in the centre of the long process. These three paths converge into junctions at the bony pedicle bridging the long process to the lenticular process. Visualisation of these paths together with the bone's absorption gives unique insights into the nanostructural arrangement. They may become relevant for modelling approaches of the human middle ear^[^ ^57^, ^58^ ^]^ or may be directly utilised in planning and optimisation of the surgery procedure at this critical junction. Given the improvements in terms of acquisition time, future studies may include a larger scope with more samples, which promotes SAS‐TT to be a viable technique for more extended studies in life science applications of statistical relevance and give access to a larger user community.

## Conflict of Interest

The authors declare no conflict of interest.

## Author Contributions

M.L., O.B., and M.W. conceived the research project. C.A., E.P., T.M., K.W., F.L., J.A.W., K.S., J.H.B., W.M.G., K.M., O.B., M.W., and M.L. planned and implemented the beamline adaptions. C.A., E.P., K.W., F.L., J.A.W., K.S., T.T., W.M.G., K.M., M.W., and M.L. carried out the X‐ray experiments. C.A., I.R.F., L.N., K.W., M.G.S., and M.L. analysed the data. L.A., A.I., M.S. and A.B. provided the sample. C.A., M.S., I.R.F., L.A., and M.L. interpreted the results. C.A., M.W., and M.L. drafted the first version of the manuscript. All authors contributed to the final version of the manuscript.

## Ethical approval

The study protocol was approved by the local ethical committee of Bern (Kantonale Ethikkommission Bern, KEK‐BE 2016‐00887) and the local ethical committee of the Paul Scherrer Institute (Ethikkommission Nordwest‐ und Zentralschweiz, 2017‐00805).

## Supporting information



Supporting Information

Supplemental Video 1

Supplemental Video 2

## Data Availability

The data that support the findings of this study are available from the corresponding author upon reasonable request.
