# Supplementary material for: Fast Small‐Angle X‐Ray Scattering Tensor Tomography: An Outlook into Future Applications in Life Sciences
Source: Small Methods. 2025 Jul 16;10(1):2500162. doi: 10.1002/smtd.202500162 (PMC12790371; doi:10.1002/smtd.202500162)
Supplement: Supplementary file 1 — Supporting Information [file SMTD-10-2500162-s002.docx]

Supporting Information:

Fast Small-Angle X-ray Scattering Tensor Tomography: An Outlook into Future Applications in Life Sciences

Christian Appel^ai^, Margaux Schmeltz^a^, Irene Rodriguez-Fernandez^ah^, Lukas Anschuetz^gj^, Leonard C. Nielsen^b^, Ezequiel Panepucci^a^, Tomislav Marijolovic^a^, Klaus Wakonig^i^, Aleksandra Ivanovic^acd^, Anne Bonnin^a^, Filip Leonarski^a^, Justyna Wojdyla^a^, Takashi Tomizaki^a^, Manuel Guizar-Sicairos^af^, Kate Smith^a^, John H. Beale^a^, Wayne Glettig^a^, Katherine E. McAuley^a^, Oliver Bunk^a^, Meitian Wang^a^* and Marianne Liebi^abe^*

a Center for Photon Science, Paul Scherrer Institute, Villigen, Switzerland

b Department of Physics, Chalmers University of Technology, Gothenburg, Sweden

c ARTORG Center for Biomedical Engineering Research, Universität Bern, Bern, Switzerland

d Department of Otorhinolaryngology, Head and Neck Surgery, Inselspital, University Hospital and University of Bern, Bern, Switzerland

e Institute of Materials, École Polytechnique Fédérale de Lausanne (EPFL), Lausanne, Switzerland

f Institute of Physics, École Polytechnique Fédérale de Lausanne (EPFL), Lausanne, Switzerland

g Department of Otorhinolaryngology, Head and Neck Surgery, Lausanne University Hospital (CHUV) and University of Lausanne (UNIL), Lausanne, Switzerland

^h Institute for Biomedical Engineering, ETH Zürich, 8092, Zürich, Switzerland^

^i Center for Scientific Computing, Theory and Data, Paul Scherrer Institute, 5232 Villigen, Switzerland^

^j The Sense Innovation and Research Center, Lausanne and Sion, Lausanne, 1007, Switzerland^

1. **Dose calculation of incus and malleus**

We calculated the dose for the incus and malleus bone. Experiments were performed at 12.4keV, with an overall flux of I_0_ = 2.56 10^12^ photons/s focussed to the spot size of the 20x20 um^2^ beam (calibration performed with glassy carbon standard, GCL14^1^). We calculate the dose for cortical bone with a density of ρ=1.92g/cm^3^, ^2^ and a mass attenuation coefficient of μ_en_/ρ=2.2m^2^/kg.^3^ For scanning SAXS with a focussed beam, the surface dose per each projection can be calculated following the equation as also used by Weinhausen et al.^4^ and Cassini et al.^5^ derived from Howells et al.^6^:

$$D=\frac{\frac{{}_{en}}{}I_{0}h ET}{xy}$$

Here, $h=12.4keV=1.986 {x 10}^{-15}J$ is the beam energy in J, $x=y$ the pixel size for fly scanning (20μm for the incus, 25μm for the malleus), ET the exposure time in seconds (83Hz for incus, 500Hz for malleus) and $\frac{{}_{en}}{}$ and $I_{0}$ as defined above. The surface dose per projection is 3.36 x 10^5^ Gy for the slow measurement of the incus, and 3.58 x 10^4^ Gy for the fast measurement of the malleus. To estimate an upper limit for the overall dose of all projections, we multiply the surface dose with the number of projections N_proj_. This results in 1.03 x 10^8^ Gy and 5.48 x 10^6^ Gy for incus and malleus respectively. Values are summarized in the table below.

| Sample | FOV  [mm x mm] | Voxel size [um] | project-ions N_proj_ | ET per projection [s] | Scan- time [h] | Total exposure | Surface dose per projection [Gy] | | Surface dose times N_proj_ |
| --- | --- | --- | --- | --- | --- | --- | --- | --- | --- |
| Incus | 1.6x2.8 | 20 | 306 | 0.012 | 16 | 13 | 3.36 x10^5^ | 1.03 x 10^8^ | |
| Malleus | 1.2x2.0 | 25 | 153 | 0.002 | 1.2 | 0.33 | 3.58 x 10^4^ | 5.48 x 10^6^ | |

Radiation dose deposited during a SAXS-TT experiment is high, which is why regular radiation damage checks are performed during the tomogram. We report about this in more detail in section 6 of the SI.

1. Transmission measurements

The transmission of the direct x-ray beam was measured on the same 2D detector (EIGER X 16M). A silicon single crystal with dimensions of 3x3.5 mm^2^ (diameter, thickness) was installed as a semi-transparent beamstop on a motorised stage in front of the detector. The direct beam footprint was measured after the semi-transparent beamstop, and its intensity was used as a measure for transmission. We note that the beam energy of 12.4 keV was chosen with sample size and dynamic range in mind to avoid over-saturating the 2D detector, or fully adsorbing the direct beam within the beamstop.

1. Reconstructions of the malleus

As mentioned in the main article, reconstructions of the malleus bone were performed on 153 projections, measured within 1.2 hours in total. We used a q range of 0.17…0.22 nm^-1^ which is still representative for the mineral particle scattering, but sufficiently low in q to ensure sufficient good photon statistics as pointed out in the analysis of the main article. For details, please check out the analysis performed in figure 2. The quality of data for the illustrated bone allows us to identify similar channels as for the incus as well as collagen the main orientation of the scattering which we can associate with the collagen fibril orientations. Q-resolved reconstructions were not performed.


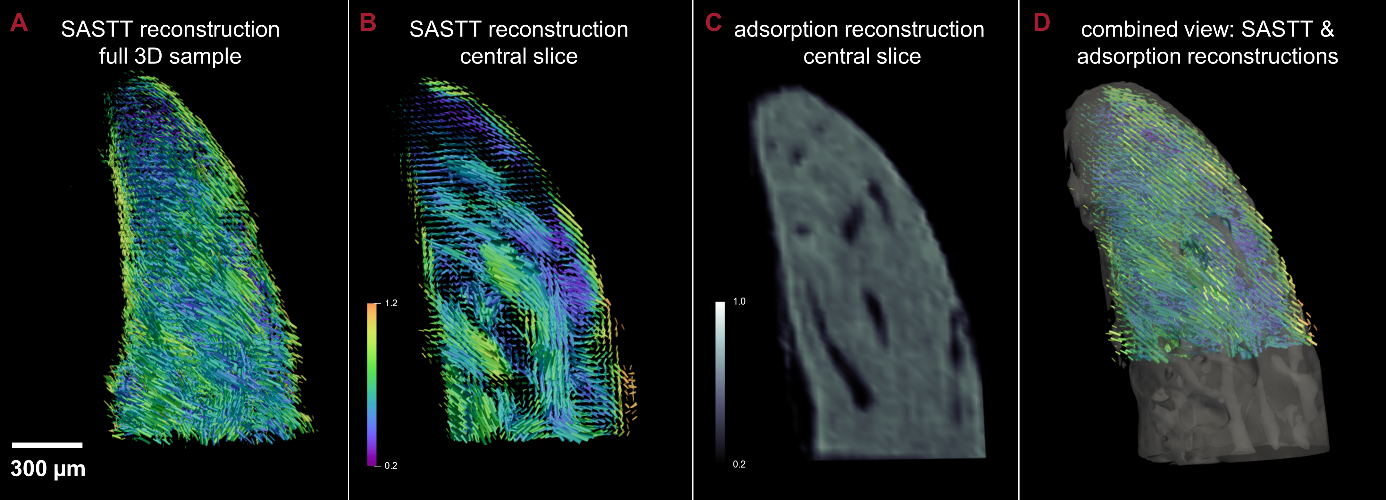


Figure S1: SAS-TT reconstructions from 150 projections of the malleus bone, acquired at a frame rate of 500 Hz within 1.2 hrs in total. The same 3D glyph presentation is used as in Figure 4 of the main manuscript. With (A) full reconstructed volume, (B) central cut through the SAS-TT reconstructions, (C) absorption data for the same cut and the combined view of isosurface rendering together with 3D glyph data in (D).

1. Key improvements for data acquisition

Scanning SAXS and SAS-TT beamlines are typically focused on versatility and rarely to optimize a single setup as the requirements to the setup may vary in-between every experiment. This makes it more difficult to optimize the acquisition to be very time efficient. On the other hand, MX beamlines are strongly focused on time efficiency but rarely change their setup in-between experimental groups. Below we list the most impactful changes to the beamline to optimize the acquisition time:

- Large area detector with fast readout ~500Hz (EIGER X).
- Scanning stages optimized for fast raster scanning (high velocity, low acceleration times). The Aerotech stages (ALS 25020) with motion overheads as little as~0.4 s in-between lines for snake motion, and ~0.58 s for a 2.5mm large sample for uni-directional motion. The malleus was scanned at 12.5 mm/s with a spatial resolution of 25 μm. According to its technical specifications, the Aerotech ALS 25020 horizontal stage supports scan speeds up to 2000 mm/s with an acceleration of 3000 mm/s². Although we have not characterized the stage performance at these limits, the specifications indicate that even faster SAXS-TT experiments are feasible with the current setup.
- SmarGon (compact goniometer) with 3 translational and 3 rotational degrees of freedom. The centre of rotation was computationally adjustable, which was extremely relevant for fast sample alignment. In future, this can be used for automated alignment.
- On-axis camera aligned with the beam. The camera combined with the SmarGon allows fast and easy sample alignment by mouse clicks. It is possible to mount a new sample and start a tomogram within 10 minutes, with the outlook for further automation in future.
- Micro-focus beam, tuneable between 5x5 µm^2^ and 80x80 µm^2^ (KB mirror pair).
- Flight tube (FT), only relevant for MX beamlines. In our case a 1.2 m FT was designed.

1. SAS-TT measurement: alignment and reconstructions

A full SAS-TT data acquisition consists of a few hundred 2D projections measured at different rotation and tilt angles, which need to be aligned before reconstruction to correct for any hard-ware misalignments. In the current case, we use the fully integrated intensity on the detector, commonly known as dark-field signal, to align all projections after the beamtime. We compute a tomogram at 0 tilt using the Filtered Back Projection (FBP) algorithm, and cross correlate all projections with the reconstruction computationally tilted by the same nominal value. The alignment shifts *dx* and *dy* are plotted in figure S2 for the incus. The repetitive motion visible for *dx* and *dy* is related to the 7 tomograms, one for each tilt angle, that were measured chronologically to sufficiently sample the 3D reciprocal space. Shifts that have been determined in this way and corrected for are smaller than 140 μm in total.

**
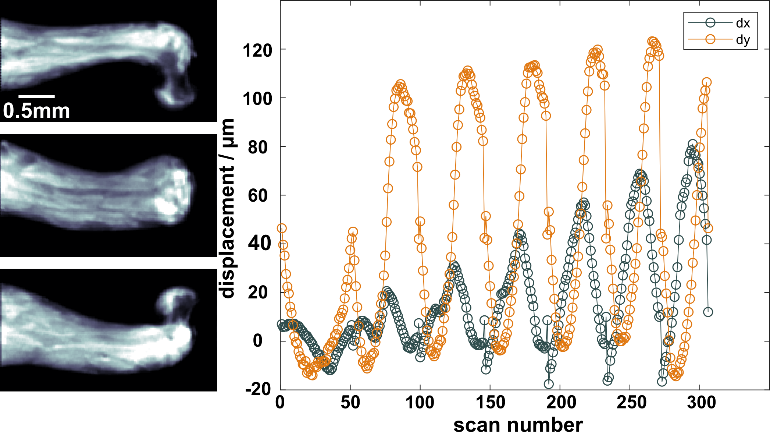
**Reconstruction of the 3D reciprocal-space map is carried out using the software package MUMOTT (*version 0.2.1*) as described in Nielsen et al. Input for the reconstruction are the azimuthal segment-wise integrated intensities for a given q. We use 100 logarithmically equally spaced q bins from the transmission corrected azimuthal data for the q-resolved reconstruction. The 3D reciprocal space in each voxel is reconstructed using band-limited Friedel-symmetric spherical functions expressed in spherical harmonics, with a band-limit of **e||** = 6. The orientation of the main intensity for each voxel is determined from the eigenvector associated with the smallest eigenvalue of the rank-2 tensor derived from the degree-2 component of the spherical function’s polynomial expansion. The robustness of the reconstruction is checked by visual comparison of 2D orientation, anisotropy, and degree of orientation between the measurements and simulated projections of the reconstructed data. The degree of orientation is calculated as the ratio between the standard deviation (anisotropic component) and mean (isotropic component).

Figure S2: 2D projections at three different rotation angles are shown for 0 ° tilt. The 1D plot shows the displacement in x and y as a function of the scan number for all tilt and rotation angles. In total, 7 full tomograms were recorded for different rotation angles.

1. **Radiation damage checks**


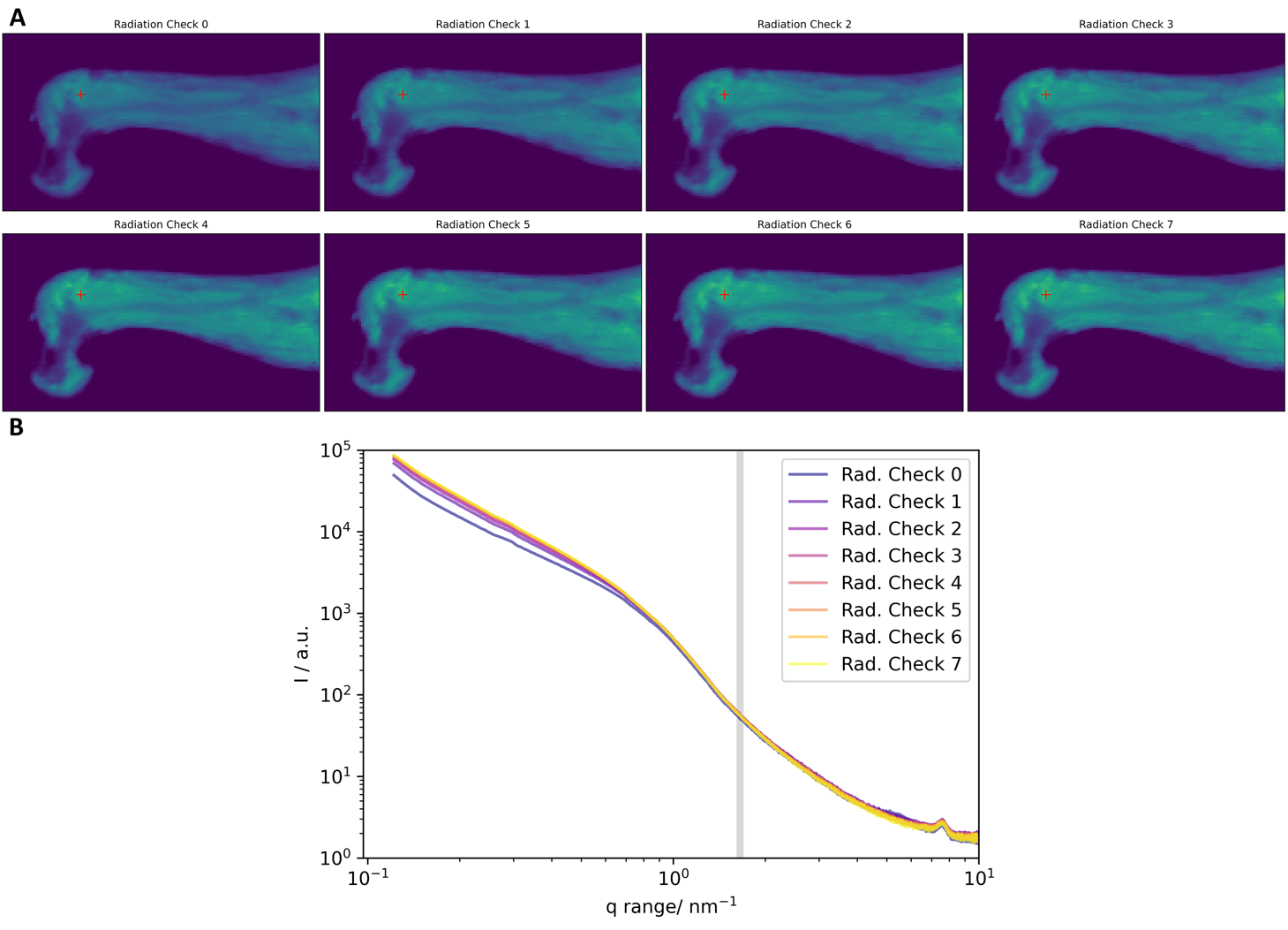
Since SAS-TT measurements are flux hungry, it is important to include regular radiation damage checks. After each tilt angle, respectively each sub-tomo, we performed a radiation damage check at zero tilt and rotation angles. This allows us to keep track of the integrity of the sample across the full measurement time. For the incus bone, we have a total of 8 measurements for 7 sub-tomograms, including one additional check at the very end of the SAS-TT measurements. Figure S3 summarises the results of these measurements. In A, we compare the integrated intensity of the full 1D scattering curve with the intensity integrated over all q; in B, we plot the full 1D scattering curve of one pixel for all projections, which is highlighted as a red cross in the projections. The data shows that at large q, no radiation damage is visible, however, we see that the power law at low q increases with increasing radiation damage check. We see the most prominent shift after the first tomogram. This region is used to determine the power law exponent which relates to the size and shape of the particle. We can deduct that the higher exponents are partially related to radiation induced changes in the nanostructure of the particles. The red box highlights the q range used for reconstruction of the reciprocal space map and orientations which are shown in Figure 4 and 5.

Figure S3: (A) Radiation damage checks measured after each sub-tomogram at zero tilt and rotation angle. All projections share the same colormap and are computed from integrating the intensity of the 1D scattering curves across the full q range. The red marker highlights a single pixel for which the 1D scattering curves are shown in (B) for all projections. The grey box highlights the region used for the reconstruction of the reciprocal space map, and thus the orientation analysis.

1. **Extracting fiber orientation from the reconstruction**

The SAXS diffraction pattern of collagen fibers is composed of two distinct signals, the meridional and equatorial (type-I) collagen signal. In detail, this is described by Giannini et al.^7^. The meridional reflections are due to the electron density periodicity along the collagen fiber axis (~65nm), whereas the equatorial scattering ones are linked to the packing of the collagen fibrils into fibers (>=100nm). Figure S4 shows the 3D reciprocal space map projected on a sphere for the meridional and equatorial scattering.

A sufficiently large q region must be chosen for proper photon count statistics, whereas the overall range should not be too large to avoid the risk of averaging out the asymmetric scattering. We checked multiple equidistant q-regions between 0.25…2.8nm^-1^ and choose 1.61…1.7nm^-1^ from within the Porod region for our data representation.The scattering from stacked mineral crystals aligns with the equatorial scattering feature of the fiber. Therefore, we can use the (equatorial-like) scattering from mineral crystals to extract the fiber orientation using the eigenvector associate with the smallest eigenvalue of the rank-2 tensor.


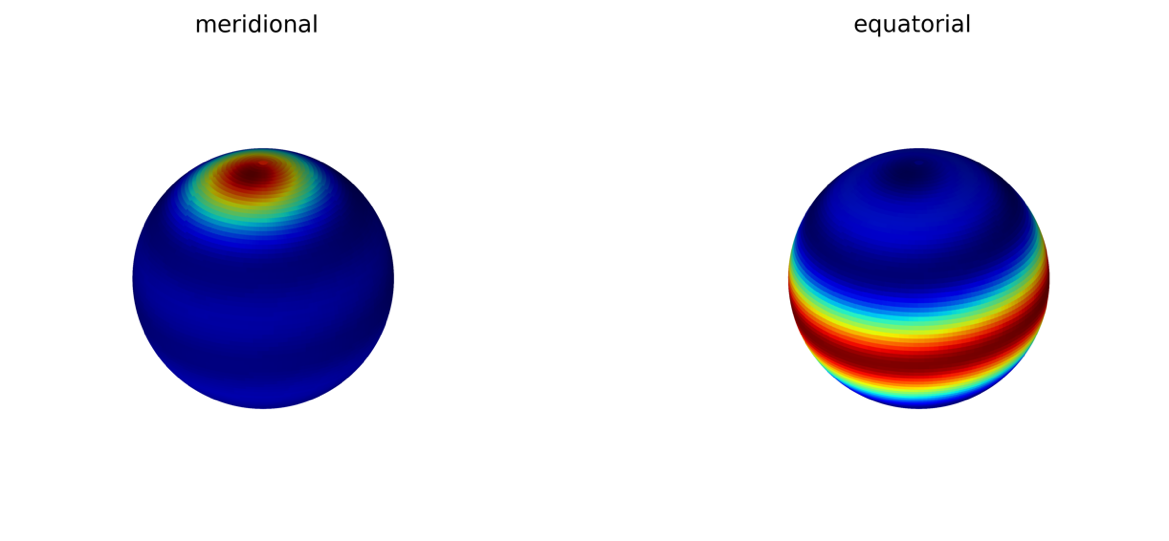


Figure S4:3D reciprocal space map projected on a sphere for simulated meridional and equatorial scattering reflections for collagen fibrils. Fiber orientation is given by the position of the meridional reflection, but can similarly be computed from the equatorial scattering.

**References:**

(1) Zhang, F.; Ilavsky, J.; Long, G. G.; Quintana, J. P. G.; Allen, A. J.; Jemian, P. R. Glassy Carbon as an Absolute Intensity Calibration Standard for Small-Angle Scattering. *Metallurgical and Materials Transactions A* **2010**, *41* (5), 1151–1158. https://doi.org/10.1007/s11661-009-9950-x.

(2) Price, G.; Biglin, E. R.; Collins, S.; Aitkinhead, A.; Subiel, A.; Chadwick, A. L.; Cullen, D., M.; Kirkby, K. J.; Schettino, G.; Tipping, J.; Robinson, A. An Open Source Heterogeneous 3D Printed Mouse Phantom Utilising a Novel Bone Representative Thermoplastic. *Phys. Med. Biol.* **2020**, *65* (10), 10NT02. https://doi.org/10.1088/1361-6560/ab8078.

(3) Seltzer, S. Tables of X-Ray Mass Attenuation Coefficients and Mass Energy-Absorption Coefficients, NIST Standard Reference Database 126, 1995. https://doi.org/10.18434/T4D01F.

(4) Weinhausen, B.; Nolting, J.-F.; Olendrowitz, C.; Langfahl-Klabes, J.; Reynolds, M.; Salditt, T.; Köster, S. X-Ray Nano-Diffraction on Cytoskeletal Networks. *New J. Phys.* **2012**, *14* (8), 085013. https://doi.org/10.1088/1367-2630/14/8/085013.

(5) Cassini, C.; Wittmeier, A.; Brehm, G.; Denz, M.; Burghammer, M.; Köster, S. Large Field-of-View Scanning Small-Angle X-Ray Scattering of Mammalian Cells. *J Synchrotron Rad* **2020**, *27* (4), 1059–1068. https://doi.org/10.1107/S1600577520006864.

(6) Howells, M. R.; Beetz, T.; Chapman, H. N.; Cui, C.; Holton, J. M.; Jacobsen, C. J.; Kirz, J.; Lima, E.; Marchesini, S.; Miao, H.; Sayre, D.; Shapiro, D. A.; Spence, J. C. H.; Starodub, D. An Assessment of the Resolution Limitation Due to Radiation-Damage in X-Ray Diffraction Microscopy. *Journal of Electron Spectroscopy and Related Phenomena* **2009**, *170* (1–3), 4–12. https://doi.org/10.1016/j.elspec.2008.10.008.

(7) Giannini, C.; Siliqi, D.; Ladisa, M.; Altamura, D.; Diaz, A.; Beraudi, A.; Sibillano, T.; De Caro, L.; Stea, S.; Baruffaldi, F.; Bunk, O. Scanning SAXS–WAXS Microscopy on Osteoarthritis-Affected Bone – an Age-Related Study. *J Appl Crystallogr* **2014**, *47* (1), 110–117. https://doi.org/10.1107/S1600576713030215.
